# Insulin-Like Growth Factors Are Expressed in the Taste System, but Do Not Maintain Adult Taste Buds

**DOI:** 10.1371/journal.pone.0148315

**Published:** 2016-02-22

**Authors:** Bradley T. Biggs, Tao Tang, Robin F. Krimm

**Affiliations:** Department of Anatomical Sciences and Neurobiology, University of Louisville, School of Medicine, Louisville, Kentucky 40202, United States of America; German Institute of Human Nutrition Potsdam-Rehbruecke, GERMANY

## Abstract

Growth factors regulate cell growth and differentiation in many tissues. In the taste system, as yet unknown growth factors are produced by neurons to maintain taste buds. A number of growth factor receptors are expressed at greater levels in taste buds than in the surrounding epithelium and may be receptors for candidate factors involved in taste bud maintenance. We determined that the ligands of eight of these receptors were expressed in the E14.5 geniculate ganglion and that four of these ligands were expressed in the adult geniculate ganglion. Of these, the insulin-like growth factors (IGF1, IGF2) were expressed in the ganglion and their receptor, insulin-like growth factor receptor 1 (IGF1R), were expressed at the highest levels in taste buds. To determine whether IGF1R regulates taste bud number or structure, we conditionally eliminated IGF1R from the lingual epithelium of mice using the keratin 14 (K14) promoter (*K14-Cre*::*Igf1r*^*lox/lox*^). While *K14-Cre*::*Igf1r*^*lox/lox*^ mice had significantly fewer taste buds at P30 compared with control mice (*Igf1r*^*lox/lox*^), this difference was not observed by P80. IGF1R removal did not affect taste bud size or cell number, and the number of phospholipase C β2- (PLCβ2) and carbonic anhydrase 4- (Car4) positive taste receptor cells did not differ between genotypes. Taste buds at the back of the tongue fungiform taste field were larger and contained more cells than those at the tongue tip, and these differences were diminished in *K14-Cre*::*Igf1r*^*lox/lox*^ mice. The epithelium was thicker at the back versus the tip of the tongue, and this difference was also attenuated in *K14-Cre*::*Igf1r*^*lox/lox*^ mice. We conclude that, although IGFs are expressed at high levels in the taste system, they likely play little or no role in maintaining adult taste bud structure. IGFs have a potential role in establishing the initial number of taste buds, and there may be limits on epithelial thickness in the absence of IGF1R signaling.

## Introduction

Chemicals in the food we eat, which characterize a substance’s taste, are detected by columnar epithelial cells on taste buds located on the tongue and palate. One of the unique features of taste buds is their extraordinary degree of plasticity. Taste cells constantly die and are renewed by a population of progenitor cells [1–3]. There are multiple types of mature taste bud cells, which differ in their anatomy, expression pattern, and function [4–7]. Therefore, progenitor cells must be able to differentiate into different specific cell types and connect to nerve fibers in order to maintain the taste bud. Many factors expressed by the surrounding epithelia, adjacent mesenchyme, and innervating nerve fibers likely support the maintenance of this dynamic system. Although a number of these factors have been identified in recent years [8], the complexity of this sensory organ makes it likely that many more remain undetermined.

Taste buds have a close trophic relationship with the nerve fibers that innervate them. For example, the neurotrophin, brain-derived neurotrophic factor (BDNF), is produced by taste bud cells and regulates both initial innervation and maintenance of innervation [9–11]. It is likely not the only growth factor involved in this function, but it is the only one that has as yet been identified. Conversely, some factors produced by neurons also serve to maintain the taste bud [12–14], but it is unclear whether these nerve fiber-derived factors participate in taste cell renewal or maintenance of differentiated taste cells. While the identities of many of these neurochemicals remain unknown, growth factors may contribute to taste bud maintenance.

Growth factors regulate the growth and differentiation of most tissues. They are commonly produced by one cell type and affect other cells by binding to a tyrosine kinase receptor. Consequently, growth factors produced by nerve fibers, mesenchyme, and adjacent epithelia potentially influence the development and maintenance of the taste system by binding tyrosine kinase receptors expressed by taste bud cells. To explore this possibility, McLaughlin [15] identified tyrosine kinases expressed at greater levels in a taste bud-enriched versus non-taste bud-enriched lingual cDNA library. Many of these tyrosine kinases are receptors, including those for epidermal growth factor, neuregulins, stem cell factors, and insulin-like growth factors (IGFs), among others [15]. Of these, the expression of IGF1R has been demonstrated in taste bud cells, and its ligands, IGF1 and IGF2, are known to be present in the surrounding nerve fibers [16]. Here, we attempt to expand upon these findings by investigating the role of IGF1R in the maintenance of taste buds.

## Materials and Methods

### Animals

Adult (P80) wild-type C57BL6/J mus musculus (n = 4) were used in real-time polymerase chain reaction (real-time PCR) experiments to determine the expression levels of growth factors and receptors in fungiform taste buds and the geniculate ganglion. *K14-Cre*::*Igf1rl*^*ox/lox*^ mice were generated by breeding *K14-Cre* mice (The Jackson Laboratory, Stock No: 018964,) with *Igf1r*^*lox/lox*^ mice (#012251). Mice were genotyped according to protocols provided by the Jackson Laboratory. All mice were housed in a central facility and maintained under controlled conditions of normal humidity and temperature, with standard alternating 12-h periods of light and darkness. Animals had free access to water and food. Mice were euthanized before fresh tissue collection or perfusion by an overdose of Avertin (2.5%, 0.04ml/gram body weight) followed by cervical dislocation. ***All animals were cared for and studied in accordance with guidelines set by the US Public Health Service Policy on the Humane Care and Use of Laboratory Animals and the NIH Guide for the Care and Use of Laboratory Animals*. *All procedures were approved by the University of Louisville Institutional Animal Care and Use Committee (IACUC)*.**

### Laser capture microdissection and RNA extraction

Taste buds and geniculate ganglion cells were isolated from adult (P80) mice via laser capture microdissection (LCM) using previously described protocols [17]. Briefly, the anterior tongue containing the fungiform field was dissected, sectioned (10 μm), and then processed to visualize taste buds. For each tongue, as many taste bud profiles as could be easily identified in unstained tissue (with a minimum of 25 taste buds/tongue) were captured on CapSure Macro LCM Caps (Molecular Devices, Sunnyvale, CA, USA). For each animal, all captured samples were stored at -80°C for RNA isolation.

Total RNA was extracted from taste buds using an RNeasy micro kit (Qiagen, Germantown, MD, USA, catalog #74004) according to manufacturer instructions. Samples underwent treatment with DNase I to eliminate all traces of DNA during the procedure. Following isolation, RNA quality was analyzed using a Bioanalyzer 2100 (Agilent Technologies, Santa Clara, CA, USA). RNA Integrity Number (RIN) and 28S/18S ratio were used to estimate RNA quality. Only RNA samples with a ratio of absorbance at 260 nm/280 nm ≥1.80 and RIN ≥8.0 were used.

### Real-time PCR

Taste bud cDNA was synthesized from total RNA using random primers (Invitrogen, Carlsbad, CA, USA). cDNA was quantified via real-time PCR using a TaqMan Universal PCR kit (Applied Biosystems, Waltham, MA, USA, catalog #4304437) and oligonucleotide primer/probe sets, which were designed from sequences available in the GenBank Database using Beacon Designer software (Premier Biosoft International, Atlanta, GA, USA). When possible, primers were chosen to span an intron to avoid genomic DNA contamination. TaqMan probes were labeled at the 5’-end with a fluorescent reporter dye (fluorescein) and at the 3’-end with a quencher dye (carboxytetramethylrhodamine). Real-time PCR reactions (Table 1) were conducted at a total volume of 10 μl, using the Master Mix and 720/200 Nm primer/probe sets (TaqMan Universal PCR kit, above) and with equal amounts of target cDNA across different time periods. All samples were run in parallel, and the 18S ribosomal RNA housekeeping gene was used to normalize for cDNA loading. Real-time PCR was performed using ABI PRISM/7900HT sequence detection systems (Applied Biosystems). For each sample, each assay was conducted in three technical replications. Real-time PCR conditions included an initial incubation at 50°C for 2 min and then 95°C for 15 min, followed by 40 cycles at 94°C for 15 s, 58°C for 30 s, and then 72°C for 30 s.

**Table 1:** Sequences of primer pairs and probes used for real-time RT-PCR.

Gene GenBank Accession #	Primers	Sequence 5’-3’	Fragment size (bp)
*Igf1r* (NM_010513)	Forward primer Reverse primer Taqman Probe	CATGCAGGAGTGTCCCTC TGAGCAGAAGTCACCGAATC TCATCCGCAACAGCACCCAGA	112
*IR* (NM_010568)	Forward primer Reverse primer Taqman Probe	GCTACATCTGATTCGAGGAGAG TGAGTGATGGTGAGGTTGTG TTGCTCCAGTCCCAGAGTTGCCT	117
*Kit* (NM_001122733)	Forward primer Reverse primer Taqman Probe	TGTGTCTACATCCGTGAACTC GTCTCCTGGCGTTCATAATTG TTGTGCTTTACCTGGGCTATGTGCTG	117
*Erbb1* (NM_007912)	Forward primer Reverse primer Taqman Probe	GGACACCCAATCAGAAAACC CATAAAGGATTGCAGACGTGG AGCTGAGAAAGACTGCAAGGCCG	85
*Erbb2* (NM_001003817)	Forward primer Reverse primer Taqman Probe	CCGGGAGTTGGTATCAGAATT TGAACGGTAGAAGGTGCTG TCATCCAGAACGAGGACTTAGGCCC	115
*Erbb*3 (NM_010153)	Forward primer Reverse primer Taqman Probe	GGGCTATGAGACGCTACTTG ACCTTAAGTTTCCTCAGCTCTG TTGTTTGCCTTCTCGCTTGGGTCC	118
*Kitl* (NM_013598.2)	Forward primer Reverse primer Taqman Probe	GCTCCCTTAGGAATGACAGC GCCAATTACAAGCGAAATGA ACTCGGGCCTACAATGGACAGCC	116
*Igf1* (NM_010512.4)	Forward primer Reverse primer Taqman Probe	GGAGACTGGAGATGTACTGTG AGTCTTGGGCATGTCAGTG TGGCGCTGGGCACGGATAGAG	89
Igf2 (NM_010512.4)	Forward primer Reverse primer Taqman Probe	TCAGTTTGTCTGTTCGGACC CACTCTTCCACGATGCCAC CTTCAAGCCGTGCCAACCGTC	87
*Nrg1* (NM_178591.2)	Forward primer Reverse primer Taqman Probe	CTTCCCTTCTCCAGCTCG CAGTCGTGGATGTAGATGTGG TTGTGGCTGAGTTCCTGACTTGGGTG	119
*Krt8* (NM_031170)	Forward primer Reverse primer Taqman Probe	TCTTCTGATGTCGTGTCCAAGTG GATCCTCGGACGGGTCTCTAG CCACTGAAGTCCTTGCCAGCCTGAGC	130
*Gapdh* (NM_008084)	Forward primer Reverse primer Taqman Probe	AATGTGTCCGTCGTGGATCTG CAACCTGGTCCTCAGTGTAGC CGTGCCGCCTGGAGAAACCTGCC	130
*β-Actin* (NM_007393)	Forward primer Reverse primer Taqman Probe	CTGGGACGACATGGAGAAGATC GTCTCAAACATGATCTGGGTCATC ACCTTCTACAATGAGCTGCGTGTGGCC	144

### Serial paraffin sectioning and taste bud quantification

Mice were perfused trans-cardially at P30 (n = 3/genotype) and P80 (n = 4/genotype) with 4% paraformaldehyde (PFA). The tongue was dissected and immersion-fixed for an additional 2 hours in 4% PFA. Whole tongues were embedded in paraffin, serially sectioned (8-μm sagittal sections), and mounted on slides, in order, without any missing sections. Sections were stained with hematoxylin and eosin (H&E) and covered with cover slips mounted with DPX (VWR, Radnor, PA, USA). Each section was examined for fungiform taste buds. Taste buds were followed across sections, such that each taste bud was recorded only once.

### Immunohistochemistry

Adult mice (P80) were trans-cardially perfused with 4% PFA (n = 3/genotype). The anterior tongue (rostral to the circumvallate papillae) was dissected and post-fixed overnight, cryoprotected with 30% sucrose, and frozen in optimal cutting temperature compound (OCT). For immunohistochemistry, the anterior tongue was sectioned (70 μm), and the sections were collected in 0.1 M phosphate-buffered saline and rinsed. After blocking with 3% normal donkey serum in 0.1 M PB containing 0.5% Triton X-100, tissues were incubated with the following primary antibodies: rat anti-cytokeratin-8 in PBS (1:50 Developmental Studies Hybridoma Bank, Iowa City, IA, USA, Cat#:Troma-1-s), goat anti-Car4 (1:500, R&D Systems, Minneapolis, MN catalog #AF2414, RRID:AB_2070332), and rabbit anti-PLCβ2 (1:500, Santa Cruz Biotechnology, catalog #sc-206, RRID:AB_632197) for 5–7 days at 4°C. After samples were incubated with primary antibodies and rinsed, the following appropriate secondary antibodies (Jackson ImmunoResearch Laboratories, West Grove, PA, USA) were applied overnight: anti-rabbit Alexa Fluor 488 (catalog #711-545-152), anti-rat Alexa Fluor 647 (catalog #712-605-153), and anti-goat Cyanine Cy3 (catalog #705-166-147). Tissues then were washed and stained with 4,6-diamidino-2-phenylindole dihydrochloride (DAPI, 2 μl in 50 ml of double-distilled H_2_O, Life Technologies, Foster City, CA, USA) for 1 hour, and mounted onto slides and cover-slipped using aqueous mounting medium (Fluoromount-G, SouthernBiotech, Birmingham, AL, USA). Serial optical sections were captured every 1 μm in labeled whole taste buds using a confocal microscope (FV1200, Olympus) under a 60X objective lens at a zoom level of 3.5. Each signal was collected separately using single-wavelength excitation and merged to produce a composite image. Ten taste buds images from the tongue tip and ten from the back of the tongue near the intermolar eminence were imaged from each animal for further analyses.

### Data analysis

Results are expressed as the mean ± standard error of the mean (SEM). For analysis of real-time PCR results, the comparative 2^-ΔΔCT^ method was used to determine target gene expression levels [18]. To measure taste bud volume, each 1-μm optical section was traced using cytokeratin 8 to define the taste bud borders and the area measured using Neurolucida imaging software (MicroBrightField, Williston, VT, USA). The area of each 1-μm optical section within the taste bud was summed to yield a value for taste bud volume. DAPI-stained nuclei within this region were counted in serial optical sections, such that each nucleus was counted once to determine the total number of cells in the taste bud. The number of PLCβ2- and Car4-positive taste receptor cells was also determined in each taste bud. The thickness of the epithelium was measured in each of 3 locations relative to the taste bud. These measures were taken from 10 taste buds per tongue region (tongue tip and back) in the same P80 *K14-Cre*::*Igf1rl*^*ox/lox*^ mice and *Igf1rl*^*ox/lox*^ mice (n = 3) that were used to quantify taste buds.

Two-way analysis of variance was used to analyze taste bud number, volume, total number of cells, and PLCβ2- and Car4-positive taste cells. Post-hoc Tukeys *t*-tests were used for individual comparisons (see S1 Table). Values of p<0.05 were considered statistically significant.

## Results

### IGF2 and IGF1R are expressed in the taste system

**Fig 1:**
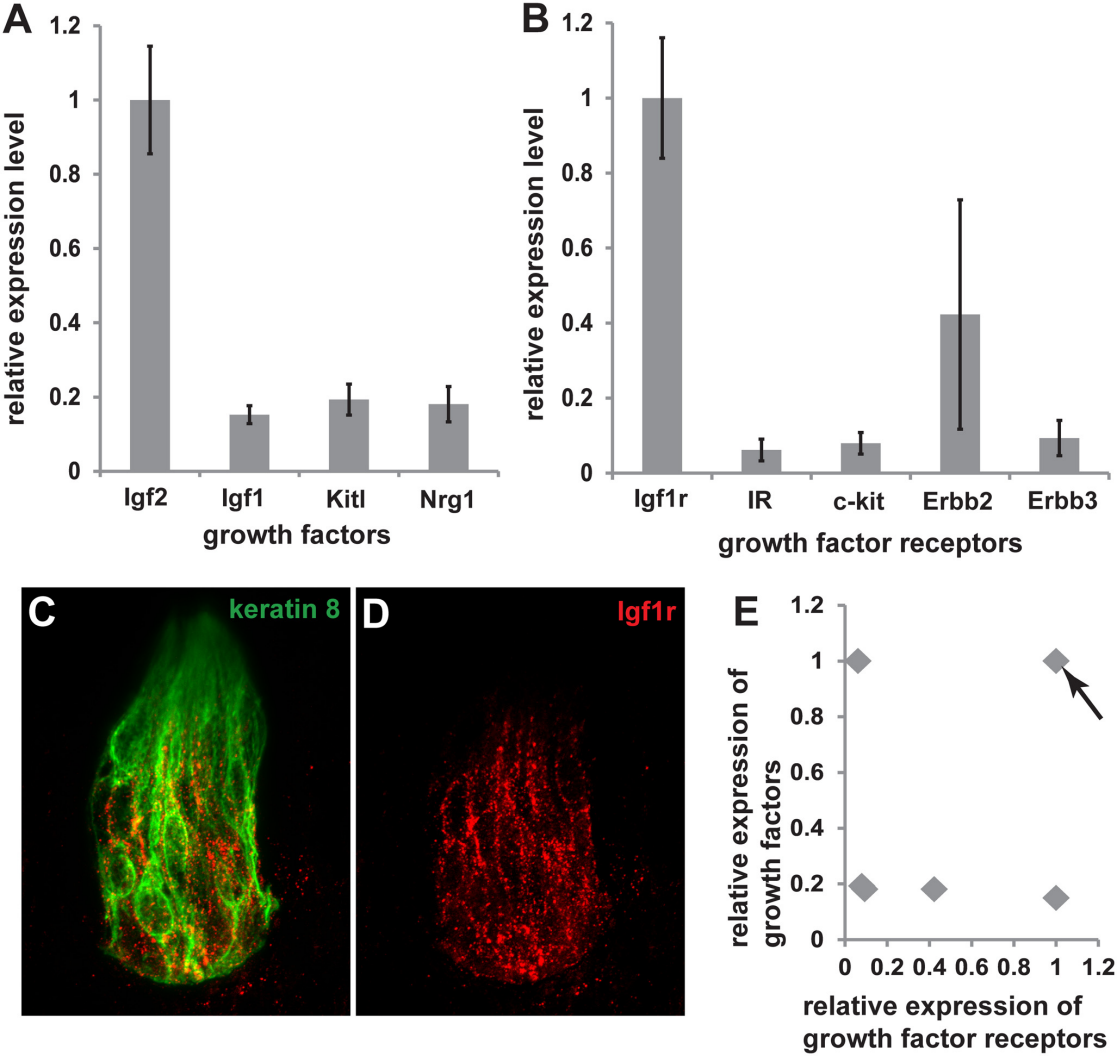
IGF2 is expressed in the geniculate ganglion, while IGF1R is expressed in taste buds. Relative expression of growth factors expressed in the geniculate ganglion (A) and receptors expressed in fungiform taste buds (B) (n = 4). IGF1R expression in the taste bud (keratin 8, green) was confirmed with immunohistochemistry (Igf1r, red). The scale bar is 20 μm for both C and D. (E) For each receptor ligand pair in (A) and (B) we plotted the relative expression of the receptor on the X-axis and the ligand on the Y-axis. IGF2 and its receptor, IGF1R, had the highest relative expression among the growth factors examined (arrow).

Growth factor receptors expressed at higher levels in taste buds than the surrounding epithelia have been previously identified [15]. We sought to identify the ligands for each of these receptors and examined their expression in the geniculate ganglion at E14.5 using previously published microarray data [19]. BDNF is expressed in the geniculate ganglion at relatively high levels and is important for taste system development [20]. We identified eight ligands (Insulin-like growth factor 2, neuregulin-3, insulin-like growth factor 1, fgf18, Kit-ligand, vascular endothelial growth factor, colony stimulating factor, neuregulin-1) whose reported expression on the microarray was higher than that of BDNF (http://www.ncbi.nlm.nih.gov/geo/query/acc.cgi?acc=GSE44734) [19]. Because factors maintaining the taste bud system would continue to be expressed in the geniculate ganglia of adult mice, we used real-time PCR to quantify the expression of all eight ligands in the adult geniculate ganglion. While four of these ligands were expressed at relatively low levels, the other four were clearly still expressed in the adult geniculate ganglion (Fig 1A).

We next verified expression of the receptors for these ligands in fungiform taste buds, and all were expressed at detectable levels (Fig 1B). The receptor expressed at the highest relative level was IGF1R (Fig 1E). We confirmed the presence of this receptor in the taste bud using immunohistochemistry, consistent with previous findings (Fig 1C and 1D) [16]. The two ligands for this receptor, IGF1 and IGF2, are both expressed in the geniculate ganglion and have been shown to be present in nerve fibers [16]. Therefore, we focused on the role of IGF1R, it being the receptor for the growth factor with the highest relative expression in the geniculate ganglion and taste buds (Fig 1E).

### Adult taste buds are not affected by elimination of IGF1R

Taste buds are maintained by certain unknown factors produced by nerve fibers [12, 14, 21]. The goal of this project was to determine if insulin-like growth factors function in this capacity. If IGF1 and/or IGF2 serve to maintain taste buds by signaling through IGF1R, then elimination of this receptor from taste buds using the *K14-Cre* construct should have similar effects on peripheral taste bud morphology. To test this hypothesis, we eliminated IGF1R using the K14 promoter (*K14-Cre*). Since precursor cells and stem cells that supply taste buds also express K14 [22, 23], this perturbation eliminated IGF1R from taste buds and the surrounding epithelium. This effect was confirmed with real-time PCR analysis of *Igf1r* expression in taste buds captured via LCM. *K14-Cre*::*Igf1r*^*lox/lox*^ mice had non-detectable levels of *Igf1r* in their taste buds (n = 3/genotype).

**Fig 2:**
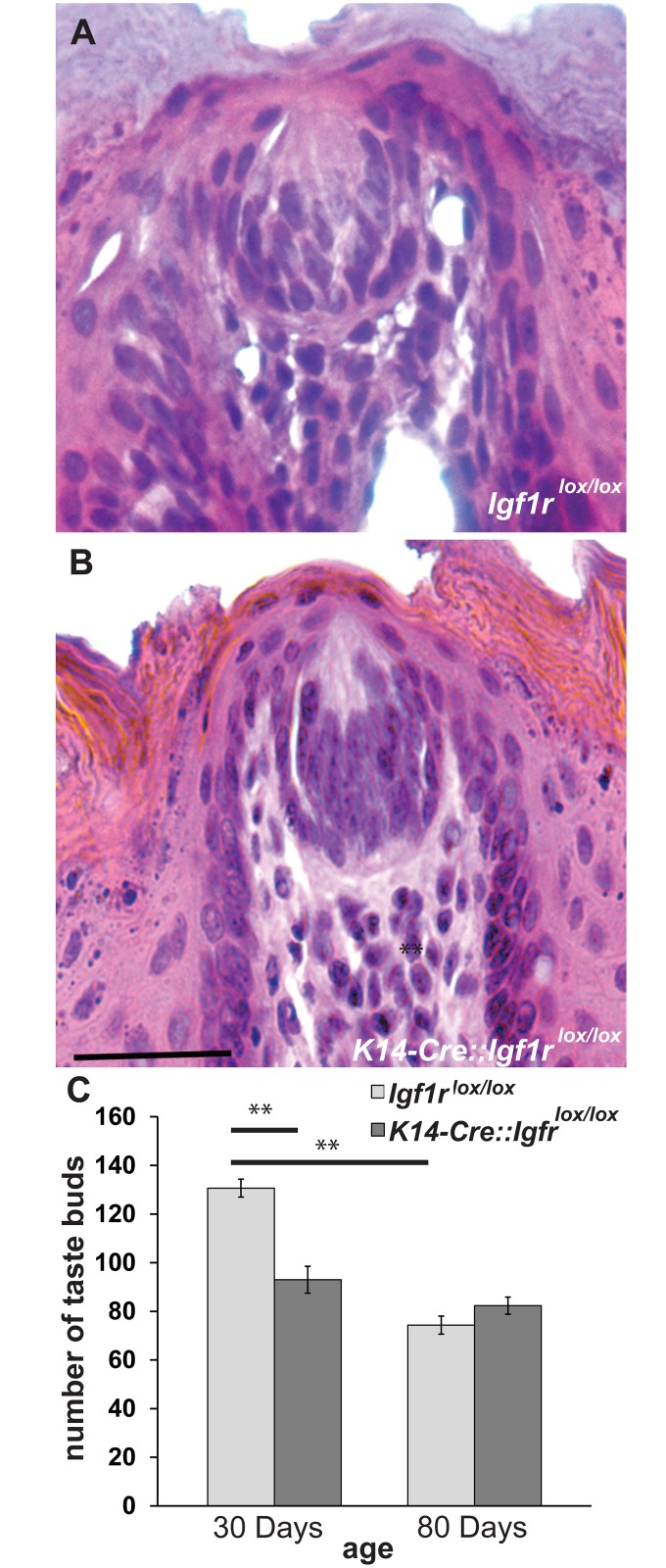
IGF1R is not required for maintenance of normal fungiform taste bud numbers in adulthood. Taste buds were counted in H&E-stained sections at P30 and P80 in *Igf1r*^*lox/lox*^ (A) and *K14-Cre*::*Igf1r*^*lox/lox*^ mice (B). There were fewer taste buds in *K14-Cre*::*Igf1r*^*lox/lox*^ mice (n = 3) than in littermate *Igf1r*^*lox/lox*^ (n = 3) at P30, (C). By adulthood (P80), taste bud numbers were identical between both genotypes (*Igf1r*^*lox/lox*^, n = 3*; K14-Cre*::*Igf1r*^*lox/lox*^ n = 4). Scale bar is 50 μm in A and B. ** p<0.01.

Taste buds were quantified in serial sections at P30 and P80 (Fig 2A and 2B) in *K14-Cre*::*Igf1r*^*lox/lox*^ mice and littermate control mice (*Igf1*r^lox/lox^). At P30, *K14-Cre*::*Igf1r*^*lox/lox*^ mice had fewer taste buds than controls (p<0.05, Fig 2C). By P80, we observed a normal post-natal reduction in fungiform taste bud number in control mice [24, 25] (p<0.01), which eliminated the difference in taste bud number between genotypes at this age. Thus, IGF1R is not required for maintenance of normal fungiform taste bud numbers in adulthood.

**Fig 3:**
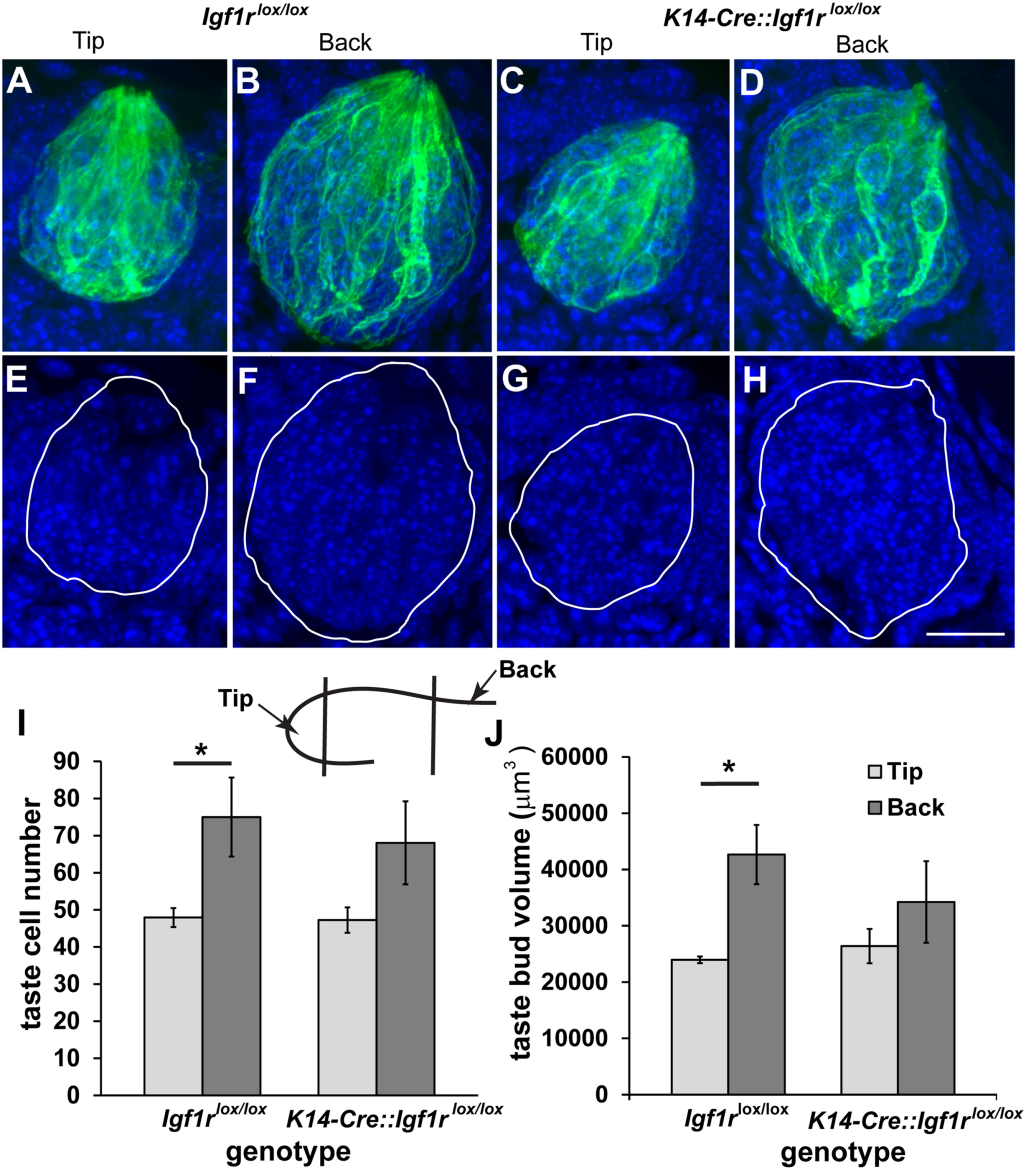
Elimination of *Igf1r* has no major effect on taste bud size. Taste buds at P80 are larger at the back of the fungiform taste field than they are at the tongue tip. The number of cells in individual taste buds and taste bud volumes were measured. The borders of taste buds were determined by staining for cytokeratin-8 (green, A-D), and cells in the taste bud were defined by a single DAPI-stained nucleus (blue, E-H). Taste buds contained more cells (I) and were larger (J) at the back of the tongue compared to the tip in *Igf1r*^lox/lox^ (n = 3 mice per region), and this difference was slightly attenuated by elimination of *Igf1r* from the tongue epithelium K14-Cre *Igf1r*^lox/lox^ (n = 3 per region). The cartoon inset of a tongue section illustrates what is meant by tip and back. The scale bar is 20 μm and applies to all panels. * p<0.05.

To quantify taste bud size and cell number, we counted the number of DAPI-stained nuclei inside taste buds, which were defined by a cytokeratin-8-stained border (Fig 3A–3H). In each animal ten taste buds from the tongue tip and ten fungiform taste buds from the back of the tongue around the intermolar eminence were captured for quantification. We also measured taste bud volume as defined by this border. In general, elimination of *Igf1r* from the epithelium had no effect on taste bud size (Fig 3I and 3J), and taste buds at the back of the tongue were substantially larger than those at the tongue tip (p<0.05 for both cell number and taste bud volume). This difference in taste bud size between the tip and back of the tongue was slightly attenuated by elimination of *Igf1r* from the epithelium.

**Fig 4:**
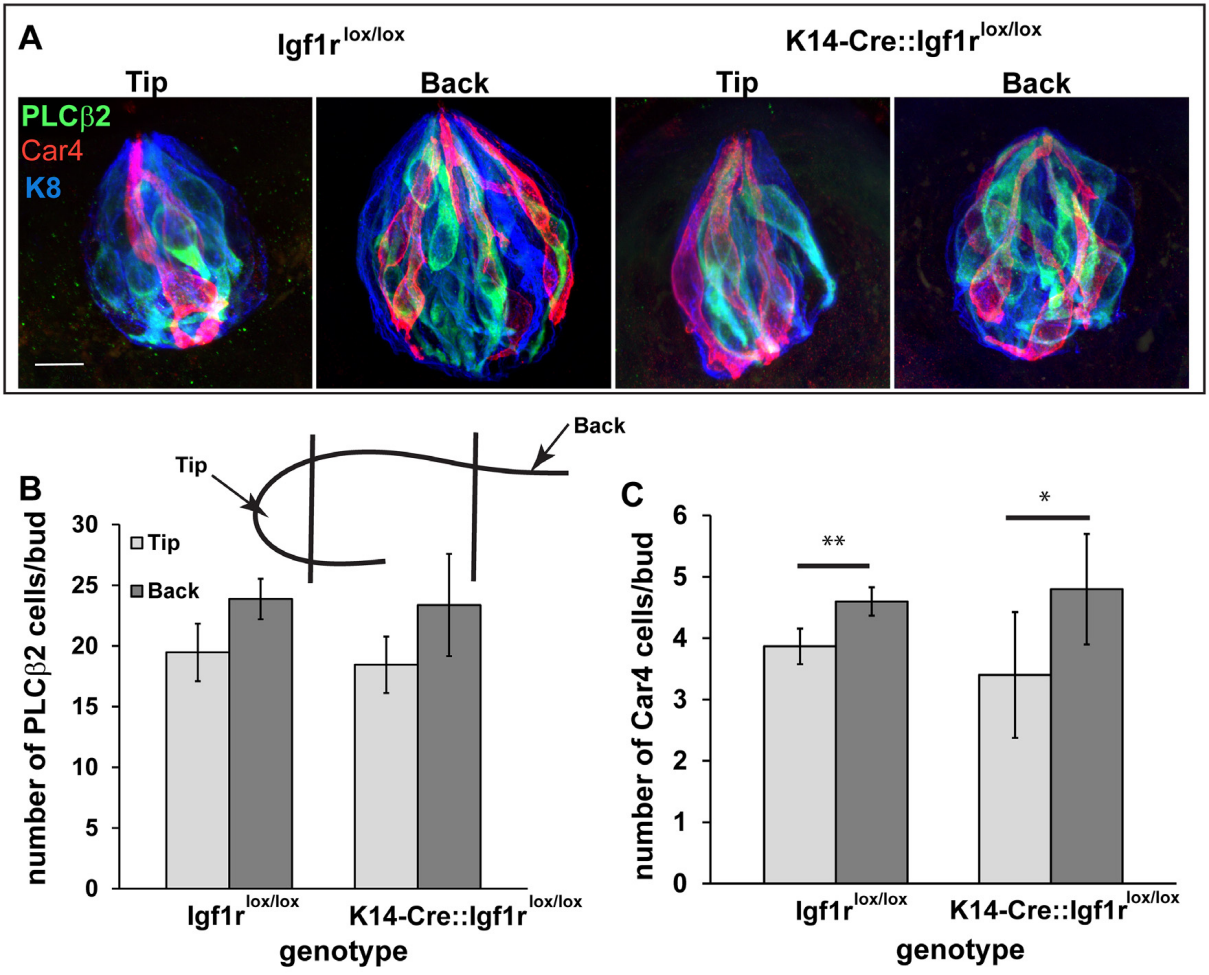
Elimination of *Igfr1* does not alter distribution of taste cells expressing PLCβ2 or Car4. Postnatal day 80 taste bud cells expressing PLCβ2 (green) and Car4 (red) were stained (A) and quantified (B, C) in cytokeratin 8 labeled taste buds (K8, blue) (n = 3 for each region and genotype). The cartoon inset of a tongue section illustrates what is meant by tip and back of the fungiform field. There was no difference in the number of PLCβ2-positive cells for either location or genotype (B). The number of Car4-positive taste buds was greater in the back of the tongue compared with the tongue tip for both genotypes (C). Scale bar is 10 μm and applies to all panels. ** p<0.01, * p<0.05.

To determine whether elimination of *Igf1r* affects the distribution of taste cell types, we counted the number of PLCβ2- and Car4-positive taste cells (Fig 4A). The two antibodies used are known to identify taste cells that transduce information via G-protein coupled receptors for sweet, bitter, and umami tastes (PLCβ2, [26]), and taste cells thought to transduce acids (Car4, [27]). There was no difference in the number of PLCβ2- and Car4-positive taste cells between *Igf1r*^*lox/lox*^ and *K14-Cre*::*Igf1r* ^*lox/lox*^ mice Fig 4B and 4C). The number of PLCβ2-positive cells did not differ across regions, while the taste buds in the back of the fungiform field contained more Car4-positive taste cells than those in the tongue tip for both genotypes (back, p<0.05; tip, p<0.01). Therefore, in addition to regional differences in taste bud size, there may also be regional differences in the distribution of different taste cell types.

### Thickness of the lingual epithelium is not affected by elimination of *Igf1r*

**Fig 5:**
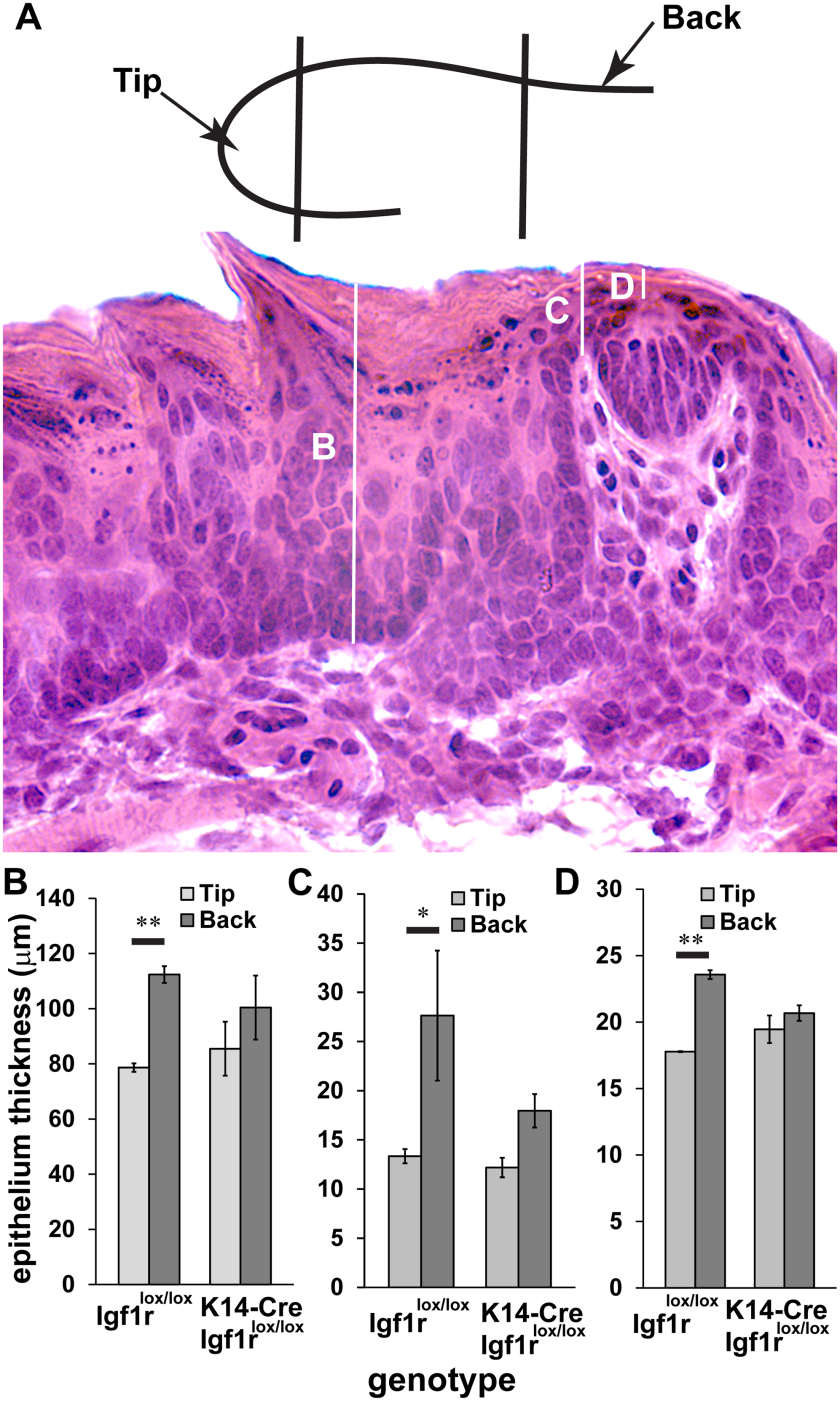
The lingual epithelium is thicker at the back than at the tip of the tongue, and this difference is attenuated by elimination of *Igf1r* from the epithelium. (A) In the samples used for taste bud quantification at P80 we measured epithelial thickness adjacent to taste buds in the tip and back portion of the tongue (10 taste buds for each location, tip vs. back, in each mouse; n = 3/genotype). The cartoon inset of a tongue section illustrates what is meant by tongue tip and back. Epithelial thickness was measured at three locations (B, C, D) with respect to each taste bud. The epithelium adjacent to the fungiform papilla (B), the taste bud (C), and dorsal to the taste bud (D) was thicker at the back portion of the tongue compared with the tongue tip. This difference was ameliorated by elimination of *Igf1r*. ***p<0.001, ** p<0.01, * p<0.05.

Taste buds extend the full depth of the lingual epithelium, and both the skin and lingual epithelium are reported to be thinner in *K14-Cre*::*Igf1r*^*lox/lox*^ mice compared with littermate control *Igf1r*^lox/lox^ mice [28]. In contrast to expectation, we found that taste buds were not smaller in *K14-Cre*::*Igf1r*^*lox/lox*^ mice. We next compared epithelial thickness at three different locations with respect to each taste bud (Fig 5A). At all three locations, there was no overall effect of elimination of *Igf1r* on epithelial thickness. Taste buds at the back of the tongue, however, were surrounded by a thicker epithelium compared with the tongue tip (Fig 5B, 5C and 5D), and this effect was abolished by elimination of *Igf1r* from the lingual epithelium.

## Discussion

The taste bud is a complex sensory end organ and numerous factors are likely involved in both its development and adult maintenance [8, 14]. Because taste cells die and are constantly replaced the maintenance of the taste bud in adulthood is an active process. Despite the abundance of factors that regulate this system both during development and adulthood [8], it is clear that not all factors which influence this system have been identified. For example, there is a reduction in taste bud number when gustatory nerves are sectioned, and those that remain are smaller in size [12, 14, 21]. These findings demonstrate that there is some unknown factor produced by nerve fibers which influences the maintenance of the taste bud. Furthermore, the number of growth factors known to influence taste system development or maintenance are limited. The neurotrophins (particularly, neurotrophin 4, and BDNF) are produced by epithelia and mesenchyme and have their primary action on the nerve fibers [11, 25, 29, 30]. Epidermal growth factor was proposed to be important in taste bud development [31], but its primary action appears to be to encourage proliferation of non-taste regions, reducing the number of fungiform papillae which contain taste buds [32]. Lastly, fibroblast growth factor (FGF) influences circumvallate papillae development [33]. Therefore, although several growth factors that influence the taste system have been examined, no growth factors that directly bind receptors in the taste bud and maintain it in adulthood have been identified, yet it does seem likely that such a factor exists.

To identify putative growth factors involved in the maintenance and renewal of taste cells, McLaughlin [15] discovered that certain growth factor receptors are expressed in taste buds at higher levels compared with the surrounding epithelium. One of these receptors, IGF1R, is expressed in fungiform and circumvallate taste buds [16]. It binds both IGF1 and IGF2, which we found in the present study to be expressed in the ganglion innervating fungiform taste buds, the geniculate ganglion. Furthermore, mice lacking IGF1R in the skin have a decreased epithelial thickness, as IGF1R regulates the proliferation of primary keratinocytes [28]. Taste cells originate from keratinocytes, and many factors known to modulate taste bud development and taste cell turnover also influence the development and maintenance of other epithelial-derived structures [34–40]. Lastly, IGF’s interact with many other factors (including Wnt’s and sonic hedgehog) [41, 42], some of which also regulate taste bud development/maintenance [8]. Because IGF1R is expressed in taste buds and plays a regulatory role in keratinocyte proliferation, we hypothesized that it might be involved in taste bud maintenance in adulthood.

This hypothesis was not supported by the present data, since by P80, we observed no effect of elimination of *Igf1r* on taste bud number, size, or cell number. Taste buds contain multiple cell types, which differ in anatomy, expression, and function [4–7, 43–48]. To verify that IGF1R was not involved in the differentiation or maintenance of specific cell types, we quantified two such cell types and found no effect of elimination of *Igf1r* from the epithelium on cell types. Collectively, these results suggest that IGF1R is not required for taste bud maintenance in adulthood.

The development of taste bud number is a dynamic process. Early post-natal animals have markedly more fungiform taste buds than they do during adulthood. Taste bud number in early development is similar to the number of embryonic fungiform papillae, and may thus be determined by initial regulation of papillae number [49, 50]. After birth, the tongue has a large number of fungiform taste buds through P20 [24, 25]. These findings are consistent with our results at P30, after which, there is a decrease in number at P60-80. It is unclear what mediates this reduction in taste bud number, although one possibility is that there is a loss or withdrawal of innervation during late post-natal development [51]. Interestingly, when we quantified taste buds at P30, which is before the postnatal loss of fungiform papillae and taste buds [24, 25], we found that elimination of *Igf1r* reduced taste bud number. This finding was in contrast to our expectations and implies that IGF1R may be involved in regulating papillae and/or taste bud number during development. Later processes that reduce taste bud number in fungiform papillae may eliminate this developmental effect.

Fungiform taste buds are typically sampled at the tongue tip to measure taste bud size because at this location, their density is highest. We sampled ten taste buds at the tongue tip and ten at the back of the tongue, near the intermolar eminence, of each animal. Fungiform taste buds at the back of the tongue were larger and contained more taste cells than those at the tongue tip regardless of genotype. The number of Car4-expressing taste cells also increased at the back of the tongue compared with the tip, while that of PLCβ2-expressing cells did not. Since Car4-positive taste cells may transduce sour stimuli [27], this difference may result in slight functional differences between the tip and the back of the tongue. If so, this finding would be consistent with human psychophysical data showing slight differences in regional sensitivities [52].

One potentially interesting effect of eliminating *Igf1r* from the epithelium was an attenuation of the increase in taste bud size at the back of the tongue. Since IGF1R has been shown to influence the proliferative capacity of keratinocytes [28], it is possible that in its absence, proliferation of taste bud precursors or stem cells is reduced. This reduction may not affect the size and cell number of most taste buds, but instead may limit the maximum size of taste buds, diminishing the difference in taste bud size between the back and the tip of the tongue. Consistent with this hypothesis, we observed that the epithelium was thicker at the back of the tongue compared with the tip, and that elimination of *Igf1r* abolishes this effect. Therefore, there appears to be a general effect of IGF1R on the keratinocyte population, which may not be specific to taste buds.

Although there exists an IGF2R, this receptor is mainly inhibitory and is involved in the degradation of IGF2 [42]. Generally speaking, IGF1 and IGF2 both exert their effects through IGF1R [41]. In the absence of IGF1R, IGF2 can bind and activate the insulin receptor [42]. Like IGF1R, the insulin receptor has been shown to be present in taste buds [16]. Therefore, it is possible that IGF2 normally modulates taste bud maintenance through activation of IGF1R, but in its absence, IGF2 may continue this modulation through the insulin receptor. To test this hypothesis, double conditional knock-out mice could potentially be generated, wherein both IGF1R and the insulin receptor are knocked out downstream of the K14-driven Cre recombinase. Unfortunately these animals die shortly after birth [28]. Since much of taste bud differentiation and maintenance occurs post-natally, information obtained from such a double conditional knock-out would presumably be limited. Despite this drawback, study of the combined effects of these two receptors on taste bud development/maintenance is warranted and may guide future studies.

## Supporting Information

S1 Table Statistical Table. (DOCX)
