# Supplementary material for: Insulin-Like Growth Factors Are Expressed in the Taste System, but Do Not Maintain Adult Taste Buds
Source: PLoS One. 2016 Feb 22;11(2):e0148315. doi: 10.1371/journal.pone.0148315 (PMC4762545; doi:10.1371/journal.pone.0148315)
Supplement: S1 Table — (DOCX) [file pone.0148315.s001.docx]

S1 Table. Statistical Table

| Figure | measure | Type of test | comparison | d.f. | F-value | p-value | Posthoc test |
| --- | --- | --- | --- | --- | --- | --- | --- |
| Figure 2 | Taste bud number | 2 X 2 ANOVA | age | 1 | 61.73 | 0.001 | Tukey HSD |
| Figure 2 | Taste bud number | 2 X 2 ANOVA | genotype | 1 | 11.22 | 0.01 | Tukey HSD |
| Figure 3I | Taste cell number | 2 X 2 ANOVA | location | 1 | 17.34 | 0.014 | Tukey HSD |
| Figure 3I | Taste bud number | 2 X 2 ANOVA | genotype | 1 | 0.30 | 0.615 | none |
| Figure 3J | Taste bud volume | 2 X 2 ANOVA | location | 1 | 7.15 | 0.05 | Tukey HSD |
| Figure 3J | Taste bud volume | 2 X 2 ANOVA | genotype | 1 | 1.07 | 0.36 | none |
| Figure 4B | PLCβ2 cell number | 2 X 2 ANOVA | location | 1 | 2.65 | 0.135 | none |
| Figure 4B | PLCβ2 cell number | 2 X 2 ANOVA | genotype | 1 | 0.07 | 0.794 | none |
| Figure 4C | Car4 cell number | 2 X 2 ANOVA | location | 1 | 17.66 | 0.145 | Tukey HSD |
| Figure 4C | Car4 cell number | 2 X 2 ANOVA | genotype | 1 | 1.72 | 0.26 | none |
| Figure 5B | lingual epithelial thickness | 2 X 2 ANOVA | location | 1 | 24.28 | 0.004 | Tukey HSD |
| Figure 5B | lingual epithelial thickness | 2 X 2 ANOVA | genotype | 1 | 3.64 | 0.115 | none |
| Figure 5C | fungiform epithelial thickness | 2 X 2 ANOVA | location | 1 | 129.51 | 0.001 | Tukey HSD |
| Figure 5C | fungiform epithelial thickness | 2 X 2 ANOVA | genotype | 1 | 0.36 | 0.36 | none |
| Figure 5D | Epithelium thickness (dorsal to taste bud) | 2 X 2 ANOVA | location | 1 | 14.21 | 0.013 | Tukey HSD |
| Figure 5D | Epithelium thickness (dorsal to taste bud) | 2 X 2 ANOVA | genotype | 1 | 2.56 | 0.17 | none |
